# Benchmarking Synolitic Graphs for Autism Classification from Multisite Resting-State fMRI

**DOI:** 10.3390/diagnostics16172746

**Published:** 2026-08-27

**Authors:** Alexey Zaikin, Daniil Vlasenko, Denis Zakharov, Janna G. Oganezova, Mikhail I. Krivonosov, Mikhail Ivanchenko, Oleg Blyuss

**Affiliations:** 1 Department of Mathematics and Women’s Cancer, University College London, London WC1E 6BT, UK; 2 Institute of Biogerontology, Lobachevsky State University, Nizhny Novgorod 603022, Russia,; 3 Institute for Cognitive Neuroscience, University Higher School of Economics, Moscow 101000, Russia; dvlasenko@hse.ru (D.V.);; 4 Academician A.P. Nesterov Department of Ophthalmology of the Institute of Clinical Medicine, Pirogov Russian National Research Medical University, Moscow 117437, Russia; 5 Centre for Cancer Screening, Prevention and Early Diagnosis, Wolfson Institute of Population Health, Queen Mary University of London, London EC1M 6BQ, UK; 6 Department of Paediatrics and Paediatric Infectious Diseases, Institute of Child’s Health, I.M. Sechenov First Moscow State Medical University (Sechenov University), Moscow 119991, Russia

**Keywords:** autism spectrum disorder, resting-state fMRI, synolitic graphs, functional connectivity, ABIDE, multisite classification, benchmark study

## Abstract

**Background/Objectives:** Synolitic graphs (SGs) were developed for task-based fMRI, where edge weights encode the discriminative power of pairwise regional features; whether similar information can be recovered from resting-state data was untested. We benchmarked SGs for autism spectrum disorder (ASD) classification using the multisite ABIDE-I dataset (871 subjects: 403 subjects with ASD, 468 typical controls; 17 sites; CC200 atlas). **Methods:** Using 5-fold cross-validation with 10 repeats and balanced accuracy as the primary metric, we compared SGs with eight baselines including correlation matrices, tangent space connectivity, elastic net logistic regression, SVM, and XGBoost. **Results:** The vectorised correlation matrix achieved 67.9% balanced accuracy (AUC = 0.743), the combined model 68.0% (AUC = 0.744), and SGs 57.0% (AUC = 0.598). SGs performed above chance (permutation *p* = 0.001), but their performance was not significantly different from that of direct logistic regression (Nadeau–Bengio *p* = 0.82). After covariate residualisation, correlation-based methods retained 66.9–67.1% balanced accuracy, while SGs achieved 55.6%. Leave-one-site-out validation yielded 66.0–66.2% for correlation-based models and 55.0% for SGs, with similar declines of approximately 2 percentage points from standard cross-validation. SG-derived ROI rankings were unstable, and ADOS severity prediction among 193 ASD participants was underpowered and inconclusive. **Conclusions:** Conventional connectivity representations substantially outperformed SGs for resting-state ASD classification, contrasting with previously reported task-fMRI advantages. No evaluated method achieved performance suitable for stand-alone clinical screening or diagnosis; this study should therefore be interpreted as a methodological benchmark rather than a validation of a clinically deployable tool.

## 1. Introduction

Autism spectrum disorder (ASD) is a neurodevelopmental condition characterised by differences in social communication and restricted, repetitive behaviours [1]. Despite decades of neuroimaging research, reliable brain-based biomarkers for ASD remain elusive [2]. Resting-state functional magnetic resonance imaging (rs-fMRI) captures intrinsic functional connectivity patterns without requiring task performance and has emerged as a promising modality for studying ASD-related brain differences [2].

The Autism Brain Imaging Data Exchange (ABIDE) initiative [3] provides a large, publicly available multisite dataset of rs-fMRI data, enabling reproducible research on ASD classification. Previous studies using ABIDE have reported classification accuracies typically ranging from approximately 67 to 70% [4,5], with substantial variability depending on methodology, preprocessing, and site selection.

A fundamental challenge in ASD classification is that the disorder is characterised by subtle, distributed brain differences rather than focal abnormalities [6]. Graph Neural Networks (GNNs) and graph-based representations have shown promise for brain connectivity analysis [7,8]. However, most approaches rely on conventional functional connectivity as the graph construction mechanism. Krivonosov et al. [9] introduced synolitic (ensemble) graphs, where edge weights are determined by the discriminative power of pairwise region features rather than correlation alone. This approach demonstrated that inter-regional synergy—task-relevant information emerging from region interactions—can be recovered even when individual regions are uninformative.

SGs were originally developed and validated for task-based fMRI, where temporal statistics carry strong task-modulated signals. Whether similar pairwise discriminative information can be recovered from resting-state temporal dynamics—which lack an explicit task structure but contain spontaneous fluctuations, quasi-periodic patterns, and intrinsic network oscillations—has not been tested. This study was designed to address this question.

In this paper, we extend the synolitic graph methodology to resting-state fMRI and the ABIDE dataset, with the following objectives:

1. Test whether SGs extract information not captured by conventional connectivity in a heterogeneous, multisite neurodevelopmental dataset.
2. Determine whether SG-derived and correlation-based patterns generalise across acquisition sites.
3. Assess whether direct and SG-derived features predict ADOS symptom severity within the ASD group.

## 2. Materials and Methods

### 2.1. Dataset and Participants

We use the ABIDE-I dataset [3], a multisite collection of resting-state fMRI data from 20 acquisition cohorts across 17 international sites. We use the C-PAC-preprocessed data available through the Preprocessed Connectomes Project [10], selecting only quality-checked subjects. The C-PAC pipeline includes slice timing correction, motion correction, nuisance regression (24 motion parameters, CompCor components derived from white matter and CSF signals, and linear and quadratic trends), band-pass filtering (0.01–0.1 Hz), and registration to MNI152 standard space. We use the filt_noglobal C-PAC derivative strategy (band-pass-filtered, without global signal regression).

The final sample comprises 871 subjects: 403 with ASD and 468 typical controls (TCs). Time series are parcellated using the CC200 atlas [11], yielding 200 functionally defined ROIs. Scan lengths vary across sites (78–316 time points, mean = 193).

### 2.2. Feature Extraction

For each subject s and ROI r, we compute temporal summary features from the resting-state time series $x_{r}^{\left(s\right)}\in R^{T}$:

$$\begin{array}{c} \mu_{r}^{\left(s\right)}=\frac{1}{T}\sum_{t=1}^{T}x_{r,t}^{\left(s\right)}\;(temporal\;mean)\\ \sigma_{r}^{\left(s\right)}=\sqrt{\frac{1}{T-1}\sum_{t=1}^{T}(x_{r,t}^{\left(s\right)}-\mu_{r}^{\left(s\right)}{)}^{2}}\;(temporal\;std) \end{array}$$

Pairwise Pearson correlation is computed: $\rho_{ij}^{\left(s\right)}=\mathrm{corr}(x_{i}^{\left(s\right)},x_{j}^{\left(s\right)})$.

### 2.3. Ensemble (Synolitic) Graph Construction

Following Krivonosov et al. [9], for each edge (i,j), a logistic regression classifier is trained on a 5-dimensional feature vector $f_{ij}^{\left(s\right)}=[\mu_{i}^{\left(s\right)},\sigma_{i}^{\left(s\right)},\mu_{j}^{\left(s\right)},\sigma_{j}^{\left(s\right)},\rho_{ij}^{\left(s\right)}]$. Edge weights encode the posterior probability difference:

$$w_{ij}^{\left(s\right)}=P(\mathrm{ASD}|f_{ij}^{\left(s\right)})-P(\mathrm{TC}|f_{ij}^{\left(s\right)})\in [-1,1]$$

Node-level features are computed by mean incident edge weight: $d_{i}=\frac{1}{N-1}\sum_{j\neq i}w_{ij}$.

To prevent data leakage, SG metatraining features are generated using out-of-fold predictions: within each outer training fold, a 4-fold inner cross-validation generates edge predictions for the metatraining participants, ensuring that the final logistic regression classifier is never trained on edge predictions from models that have already seen those participants.

### 2.4. Comparator Methods

We compare SGs against eight methods:

Direct (LogReg): Logistic regression on $\left[\mu_{1},\sigma_{1},\ldots ,\mu_{200},\sigma_{200}\right]$ (400 features).

Corr Matrix (LogReg): Logistic regression on the vectorised upper-triangle correlation matrix (19,900 features).

Corr Graph (LogReg): Z-scored correlation graph with node summaries.

Tangent (LogReg): Tangent space connectivity [12], which was calculated using a fixed subset comprising the first 50 CC200 ROIs in atlas order (indices 0–49). This subset was chosen a priori for computational feasibility and was not selected using participant data or diagnostic labels. Within each outer cross-validation fold, the tangent space reference covariance and subsequent feature standardisation were estimated using the training data only and then applied to the held-out test data.

Elastic Net: Elastic net logistic regression (*ℓ*_1_/*ℓ*_2_ = 0.5) on direct features [13].

SVM: Calibrated linear SVM on direct features [14,15].

XGBoost: Gradient-boosted trees on direct features [16].

Combined (LogReg): Logistic regression on concatenated means, stds, and vectorised correlations.

### 2.5. Validation Strategy

#### 2.5.1. Primary Validation

All methods use 10 repeats of 5-fold stratified cross-validation with distinct random seeds [17]. Balanced accuracy is the primary metric [18], with a balanced accuracy chance level of 50%. We additionally report AUC, sensitivity, specificity, F1, and PR-AUC with mean ± std across all 50 evaluations.

Statistical comparisons use the Nadeau–Bengio-corrected repeated cross-validation test [19], which adjusts the variance estimate for the non-independence of overlapping training sets:

$${\hat{\sigma }}_{\mathrm{corrected}}^{2}=\left(\frac{1}{n}+\frac{n_{\mathrm{test}}}{n_{\mathrm{train}}}\right){\hat{\sigma }}_{d}^{2}$$

where n is the number of fold evaluations, $n_{\mathrm{test}}/n_{\mathrm{train}}$ is the test-to-train ratio, and ${\hat{\sigma }}_{d}^{2}$ is the sample variance in the paired differences.

#### 2.5.2. Site-Aware Validation

Leave-one-site-out (LOSO): For each site with ≥20 subjects, train on all other sites, and test on the held-out site. The results are reported as the unweighted mean across sites.

Site-stratified group CV: This consists of 5-fold stratified group cross-validation using site as the grouping variable.

Both LOSO and site-stratified validation are evaluated for all five principal methods: direct, correlation matrix, combined, tangent, and SGs.

#### 2.5.3. Permutation Test

A total of 1000 permutations with shuffled diagnosis labels establish the null distribution. The corrected empirical *p*-value uses $p=(n_{\mathrm{exceed}}+1)/(n_{\mathrm{perm}}+1)$ [20].

### 2.6. Confounding and Sensitivity Analyses

Phenotype-only baseline: As an exploratory analysis, logistic regression using age, sex and full-scale IQ was evaluated in the 811 participants with complete data; no imputation was performed. Full-scale IQ was missing for 60 participants (6.9%): 27 of 403 ASD participants (6.7%) and 33 of 468 TC participants (7.1%). The missingness rate did not differ between diagnostic groups (χ^2^ = 0.04; *p* = 0.84) and was concentrated at specific sites, consistent with site-level differences in data availability. Thus, we found no evidence of differential IQ missingness by diagnosis. The imaging models were evaluated in the full sample of 871 participants; consequently, the phenotype-only and imaging performance estimates are reported descriptively and are not treated as directly comparable.

Covariate residualisation: Imaging features are residualised for age, sex, and mean framewise displacement [21] within each training fold. Residualisation is performed separately for all five principal methods.

Scan duration: Analysis was restricted to subjects above the 25th percentile scan length.

### 2.7. Implementation Details

A base random seed of 42 was used for reproducibility; each of the 10 CV repeats used a distinct random state (seeds 42 through 51), producing different stratified fold partitions, with all methods receiving the same fold assignments within each repeat. Logistic regression used C = 1.0, L2 regularisation, the LBFGS solver and max_iter = 200; StandardScaler was fitted on the training data only. Hardware: Apple M4 Max (Apple Inc., Cupertino, CA, USA), 16 cores (12 used), arm64. Software: Python 3.13.1 (Python Software Foundation, Beaverton, OR, USA); scikit-learn 1.8.0 (logistic regression, elastic net, SVM, cross-validation) [22], NumPy 2.4.3; nilearn 0.14.0 [23], XGBoost 3.2.0 (XGBoost Developers) [16].

### 2.8. Ablation Analysis

To isolate the contribution of each feature type to classification performance, we evaluated models trained on temporal means only, temporal standard deviations only, the vectorised correlation matrix only, and the combined (mean + standard deviation + correlation) feature set, each assessed with the same 10 repeats × 5-fold stratified cross-validation as the primary analysis. For SGs, we additionally compared variants restricted to positive-only, negative-only, and absolute edge weights and evaluated balanced accuracy as a function of the number of ROIs included in the ensemble graph.

### 2.9. Nested ROI Selection

Within each outer training fold, individual ROIs were ranked according to their mean classification accuracy obtained through inner cross-validation using the training data only. The ROIs were split at the median into fold-specific top and bottom halves of 100 ROIs each. Separate SG models were then fitted using these two subsets and evaluated on the corresponding held-out outer test fold. Reported top-half and bottom-half performance was aggregated across all 50 outer fold evaluations. Separately, ranking stability was assessed using Spearman rank correlations between fold-specific rankings and by recording the proportion of evaluations in which each ROI appeared in the top or bottom half. ROIs appearing in the same half in more than 80% of evaluations were described as consistently selected; these consistency frequencies were not used to define the classification subsets.

### 2.10. ADOS Severity Prediction

Among the 193 participants with ASD with available ADOS Gotham Calibrated Severity Scores [24], ridge regression with a fixed regularisation parameter of α = 1.0 was used to predict symptom severity from either direct features or SG node features. No hyperparameter tuning was performed. Models were evaluated using 10 repeats of five-fold cross-validation, with feature standardisation fitted using the training data only within each fold. Performance was summarised using Pearson’s correlation coefficient, the coefficient of determination (R^2^) on held-out observations, and mean absolute error (MAE). A training fold mean predictor, which assigned the mean severity score from the training fold to every participant in the corresponding test fold, was used as a null baseline.

## 3. Results

### 3.1. Cohort Characteristics

**Figure 1:**
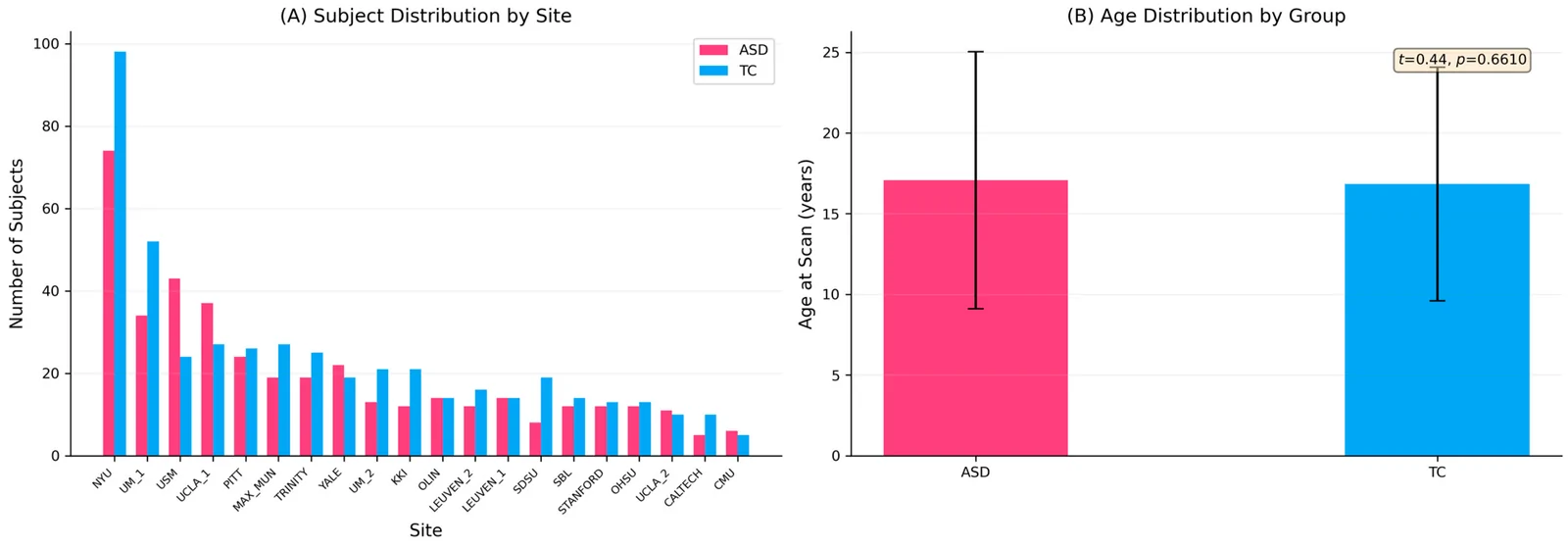
(**A**) Subject distribution across ABIDE-I sites. (**B**) Age by diagnostic group.

Data were collected from 20 acquisition cohorts across 17 sites (Figure 1, Table 1), with sample sizes ranging from 21 (UCLA_2) to 172 (NYU).

### 3.2. Primary ASD Classification

**Figure 2:**
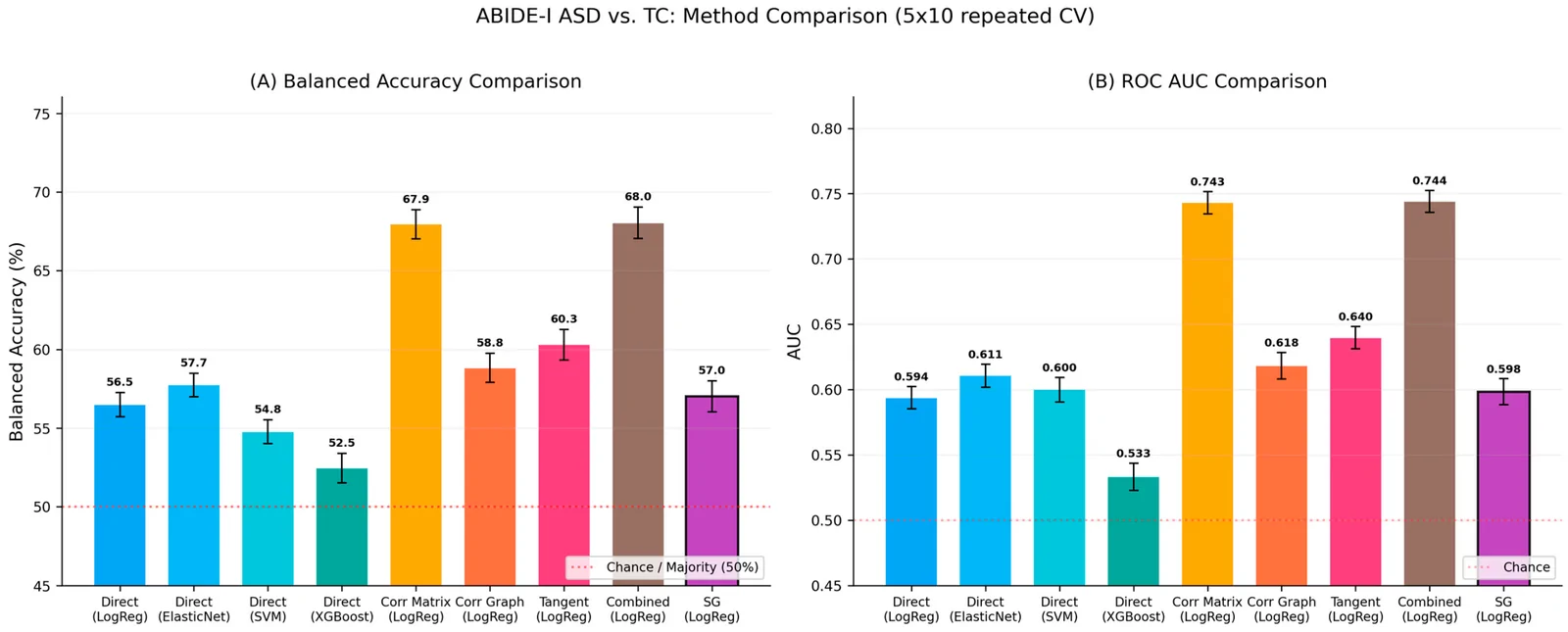
(**A**) Balanced accuracy; (**B**) AUC comparison. Error bars correspond to 95% CI. Dashed line: Balanced accuracy chance level (50%).

Table 2 and Figure 2 present the results for all nine methods, each evaluated with 10 repeats × 5 folds = 50 evaluations.

The vectorised correlation matrix and combined features achieve the highest performance (balanced accuracy ≈68%, AUC ≈ 0.74). SGs achieve 57.0% balanced accuracy—not significantly different from that of direct logistic regression (+0.5 pp, Nadeau–Bengio p=0.82) but significantly below that of the correlation matrix (−10.9 pp, p<0.001) and combined model (−11.0 pp, p<0.001).

### 3.3. Permutation Test

**Figure 3:**
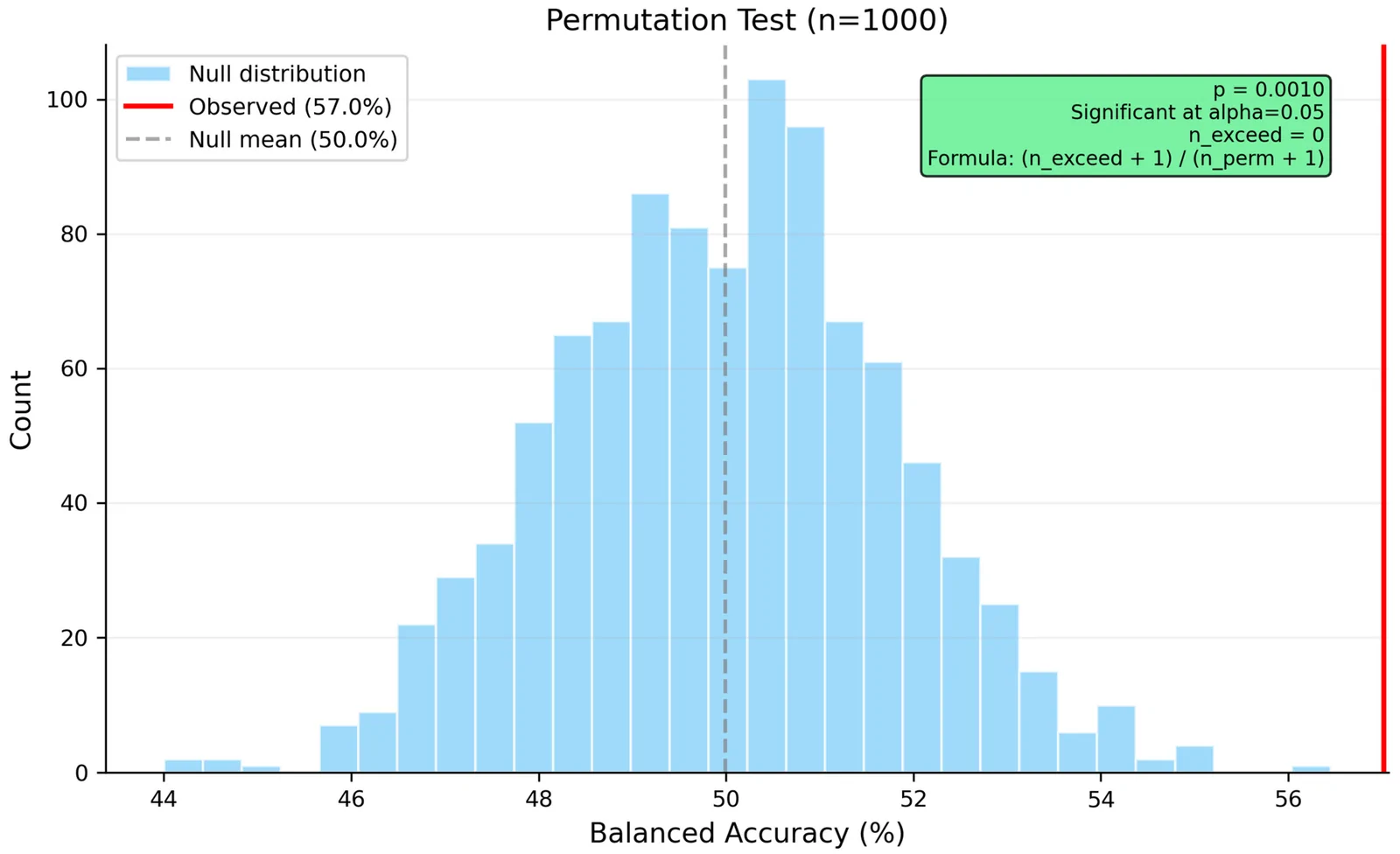
Permutation test: Null distribution (1000 permutations) versus observed SG balanced accuracy. Corrected p=0.001.

The permutation test (1000 permutations) yields a null distribution with mean 50.0 ± 1.8%. The observed SG performance (57.0%) exceeds all null values ($n_{\mathrm{exceed}}=0$), giving a corrected empirical p=(0+1)/(1,000+1)=0.001 (Figure 3).

### 3.4. Site-Aware Validation

Table 3 presents site-aware validation for all five principal methods.

**Figure 4:**
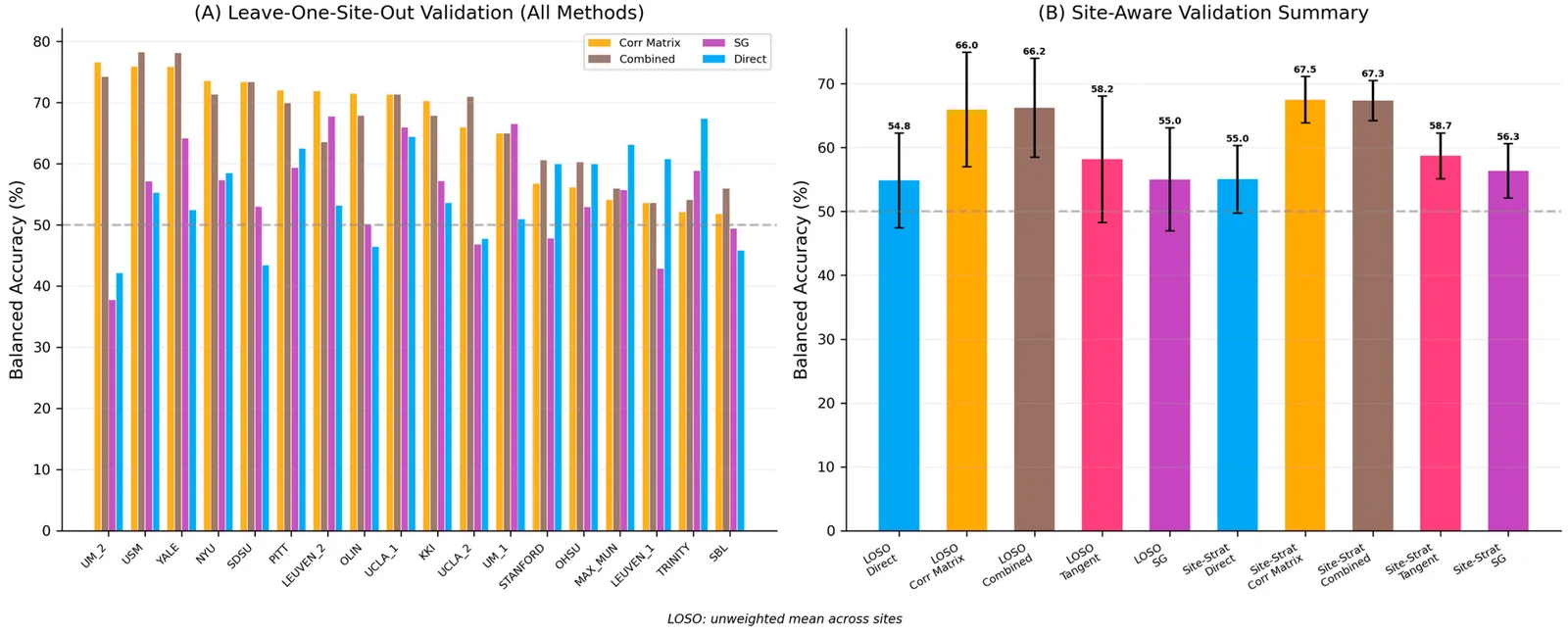
(**A**) Per-site LOSO balanced accuracy for all methods. (**B**) Aggregate site-aware validation.

Under LOSO validation, correlation-based models retained substantially higher absolute balanced accuracy (66.0–66.2%) than SGs (55.0%). However, the decline from standard cross-validation was similar for the correlation-based models and SGs (approximately 1.8–2.0 percentage points), providing no evidence of a uniquely larger site-related performance loss for SGs. Figure 4 shows the per-site results.

### 3.5. Ablation Analysis

**Figure 5:**
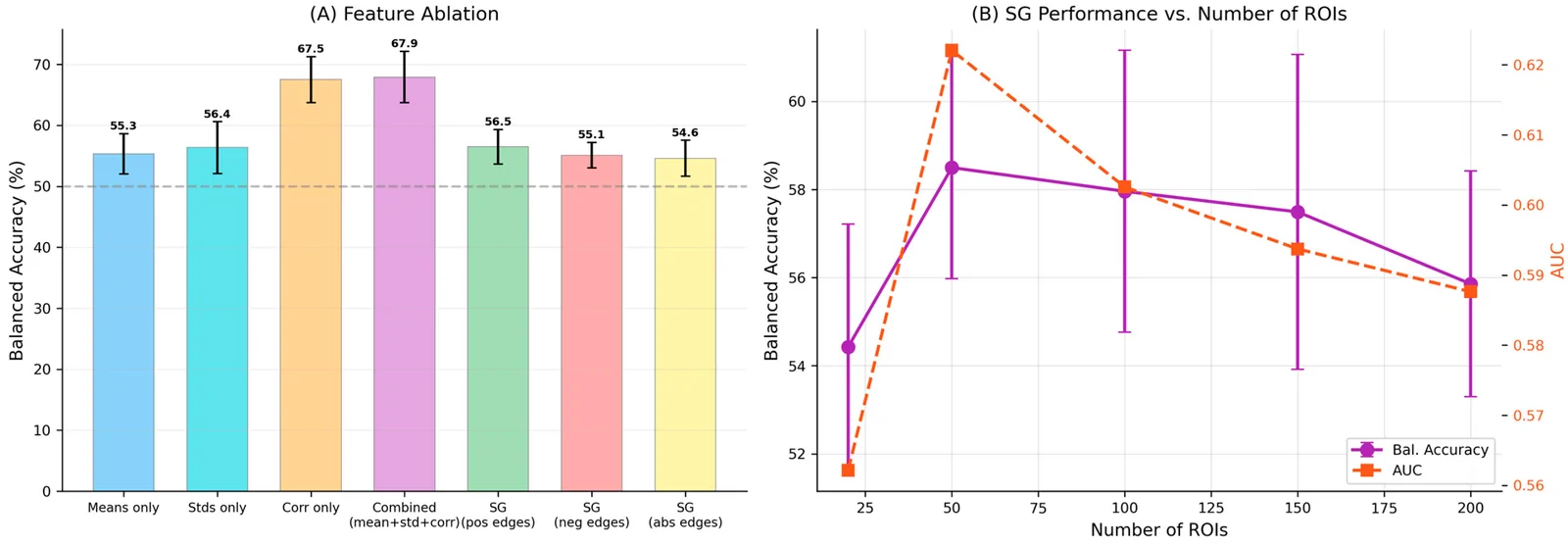
(**A**) Feature ablation. (**B**) SG balanced accuracy as a function of the number of ROIs.

The ablation results (Figure 5) show:

- Correlation features alone (67.5%) explain nearly all discriminative performance, dominating means (55.3%) and standard deviations (56.4%).
- SG performance peaks at approximately 50 ROIs (58.5%) and declines with additional ROIs, suggesting that adding weakly informative ROIs introduces noise.
- Positive edge (56.5%) and negative edge (55.1%) SG variants both carry information, with positive edges performing slightly better.

### 3.6. Nested ROI Selection

**Figure 6:**
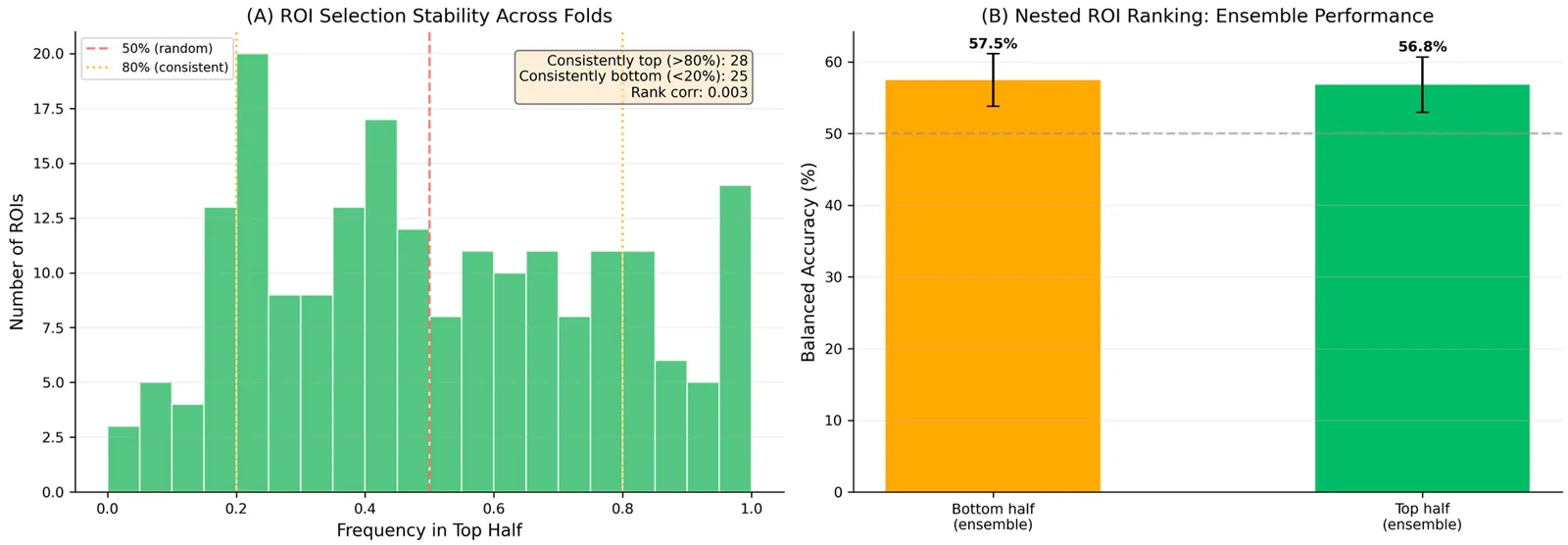
(**A**) Frequency with which each ROI appeared in the fold-specific top half across cross-validation evaluations. (**B**) The balanced accuracy of SG models using top-half and bottom-half ROI subsets selected independently within each outer training fold.

ROI rankings showed near-zero stability across training samples, with a mean Spearman correlation of 0.003 across fold-specific rankings. Twenty-eight ROIs appeared in the top half and 25 appeared in the bottom half in more than 80% of evaluations. These frequencies were used only to describe ranking stability. The classification models used top-half and bottom-half ROI subsets selected separately within each outer training fold. The resulting balanced accuracies were 56.8% for the top-half subsets and 57.5% for the bottom-half subsets (Figure 6), indicating no meaningful performance advantage for the relatively higher-ranked ROIs.

### 3.7. ADOS Severity Prediction

Among 193 participants with ASD with ADOS Gotham Calibrated Severity Scores [24] (mean = 7.0 ± 2.1, range 1–10), neither direct features (r = −0.073; R^2^ = −2.14) nor SG node features (r = −0.033; R^2^ = −3.79) predicted symptom severity. Both methods performed substantially worse than a simple training fold mean predictor (MAE = 1.68; R^2^ ≈ 0). The strongly negative R^2^ values indicate predictions worse than predicting the mean. However, with only 193 participants with ASD and high-dimensional features, this analysis was underpowered for detecting small-to-moderate effects, and the negative result should be interpreted as inconclusive rather than as definitive evidence of no signal.

### 3.8. Confounding and Sensitivity Analyses

**Figure 7:**
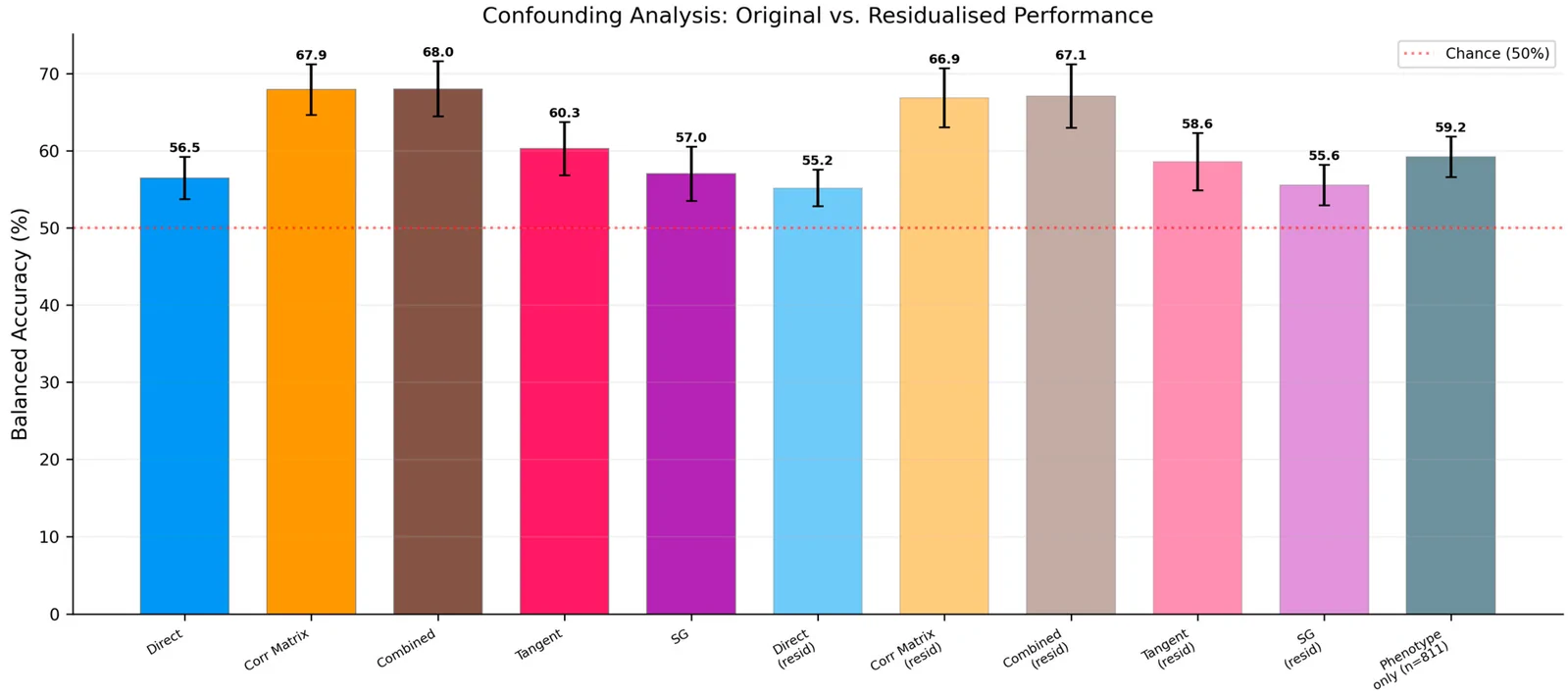
Original and covariate-residualised performance for the imaging models, with the exploratory phenotype-only baseline shown separately. The phenotype baseline was evaluated in 811 complete cases, whereas imaging models were evaluated in 871 participants.

Table 4 and Figure 7 summarises confounding analyses.

Key findings: The exploratory phenotype-only model achieved 59.2% balanced accuracy among the 811 participants with complete age, sex and full-scale IQ data. Because the imaging models were evaluated in all 871 participants, this estimate is reported descriptively and should not be interpreted as a direct comparison with SG or the other imaging models. After residualisation for age, sex and mean framewise displacement within each training fold, balanced accuracy decreased from 67.9% to 66.9% for the correlation matrix model and from 68.0% to 67.1% for the combined model. SG performance decreased from 57.0% to 55.6%. These findings suggest that age, sex and motion do not explain most of the discrimination achieved by the correlation-based models.

The phenotype-only result should be interpreted with caution: sites differ in their age and sex distributions and ASD-to-TC ratios, so the phenotype model may partly exploit site identity through demographic proxies. The more informative test is the residualisation analysis, which shows that the imaging-based correlation models retain strong performance (66.9–67.1%) after removing the linear effects of age, sex, and motion within each training fold.

## 4. Discussion

This study provides a comprehensive benchmark of synolitic graphs for autism classification in the multisite ABIDE-I dataset. The principal findings are:

1. SGs achieved statistically significant but modest above-chance discrimination. With 57.0% balanced accuracy and a corrected permutation *p* = 0.001, SGs performed above chance. However, the improvement over direct logistic regression was only 0.5 percentage points and was not statistically significant (*p* = 0.82, Nadeau–Bengio-corrected test). Elastic net logistic regression achieved slightly higher balanced accuracy than SGs using the direct feature set.

2. Conventional connectivity representations substantially outperformed SGs. The vectorised correlation matrix (67.9%) and combined model (68.0%) achieved ∼11 percentage points higher balanced accuracy than SGs (*p* < 0.001). This contrasts with the task-fMRI findings where SGs showed clear advantages [9], suggesting that in resting-state data, discriminative information resides primarily in the correlation structure rather than in the SG edge representation.

3. Correlation-based discrimination remained substantially stronger than SGs under site-held-out validation. Leave-one-site-out balanced accuracy was 66.0–66.2% for the correlation-based models compared with 55.0% for SGs. The absolute decreases from standard cross-validation were similar in magnitude (approximately 1.8–2.0 percentage points), so these results do not establish that SGs had a uniquely larger site-induced performance drop. One plausible explanation for its lower LOSO performance is that SGs train many pairwise classifiers on edge features within each training fold; acquisition-related shifts in temporal means, standard deviations or correlations may therefore affect the calibration of learned edge weights at a held-out site. Pearson correlation was scale-normalised, and the final correlation matrix classifier was globally L2-regularised, which may offer greater robustness. This mechanism was not directly tested and should be investigated in future work.

4. The exploratory phenotype-only model achieved 59.2% balanced accuracy in 811 complete cases, but because it was evaluated in a different sample, its performance is not directly comparable with the imaging models. After within-fold residualisation for age, sex and motion, the correlation-based methods lost only ∼1 percentage point, suggesting that these measured covariates do not explain most of their discrimination.

5. ADOS severity prediction was inconclusive. Neither direct nor SG features predict ADOS severity among the 193 ASD participants (R^2^ < 0). However, no a priori power calculation was performed, and the sample size is insufficient for detecting small effects in high-dimensional cross-validated prediction. This result should not be interpreted as definitive evidence that resting-state features carry no severity information; rather, substantially larger ASD cohorts with standardised severity assessments would be needed to address this question.

Our best performance (68.0% balanced accuracy, AUC = 0.744) using combined features is consistent with previous ABIDE studies. Abraham et al. [4] reported ∼67% using tangent connectivity; Heinsfeld et al. [5] achieved 70% using deep learning on correlation matrices. The consistent finding is that functional connectivity—particularly when used with regularised classifiers—captures the primary ASD-related signal in resting-state fMRI.

The performance levels reported here are insufficient for clinical screening or diagnosis. There is no single AUC or sensitivity/specificity threshold that defines clinical utility; the required performance depends on the intended clinical role, disease prevalence, downstream testing and the consequences of false-positive and false-negative results. The USPSTF has also highlighted uncertainty regarding the balance of the benefits and harms of population ASD screening in young children without prior clinical concern [25]. With a sensitivity of approximately 65% and AUC = 0.744, our best model would miss a substantial proportion of ASD cases and is not suitable as a stand-alone screening or diagnostic tool. The present work should therefore be understood as a methodological benchmark comparing connectivity representations rather than as a validation of a clinically deployable tool.

The key insight is that SGs’ edge-level compression—reducing five pairwise features to a single scalar weight via local logistic regression—discards information that is preserved when the full correlation matrix is passed directly to a globally regularised classifier. This explains the performance gap: if the ASD-related signal is predominantly linear in the 19,900 pairwise correlations, the L2-regularised logistic regression on the full matrix is more expressive than a model operating on 200 SG-derived node summaries. This outcome was not predictable a priori, because in task-fMRI settings, the SG approach outperformed global classifiers [9], suggesting that the non-linear edge weighting captured synergistic information absent from raw correlations. The present results demonstrate that this advantage does not transfer to the resting-state setting, where temporal statistics carry limited discriminative signal (ablation: means 55.3%; standard deviations 56.4% vs. correlations 67.5%).

Several methodological points emerge.

Unified validation is essential. Using the same number of repeats for all methods enables valid paired comparisons. The Nadeau–Bengio-corrected test [19] is preferred over the standard paired *t*-test for repeated CV.

Balanced accuracy matters. With 46.3% ASD prevalence, the majority-class predictor achieves 53.7% raw accuracy but only 50% balanced accuracy. This distinction matters for methods with extreme sensitivity/specificity asymmetry (e.g., SVM: 87.5% specificity, 22.1% sensitivity).

Site-aware validation is critical for the best-performing models. Demonstrating that the highest-performing methods (not just SGs) generalise across sites is essential for clinical relevance.

Both positive edge (56.5%) and negative edge (55.1%) SG variants carried modest discriminative information. In the SG framework, however, edge sign refers to the direction of class evidence: positive weights indicate that the five-dimensional pairwise feature vector favours ASD classification, whereas negative weights favour TC classification. Because this feature vector includes temporal means, temporal standard deviations and correlation, SG edge sign should not be interpreted directly as positive versus negative functional connectivity or as hyperconnectivity versus hypoconnectivity. The result is compatible with the broader literature reporting both increased and decreased functional connectivity in ASD [2,3], but the present ablation does not directly test that biological distinction.

XGBoost achieved only 52.5% balanced accuracy on the 400-dimensional direct feature set. A plausible explanation for this is that in this high-dimensional and weak-signal setting, boosted trees may fit noisy or unstable splits, whereas L2-regularised logistic regression is well suited to signals distributed diffusely across many weakly informative features. However, the present result concerns the XGBoost implementation evaluated here and should not be interpreted as evidence that tree-based methods are generally unsuitable for ASD classification, because their performance can depend materially on model specification and hyperparameter choice.

The near-zero ROI ranking stability (mean Spearman ρ = 0.003) indicates that the fine-grained ordering of individual ROIs is not replicable across data partitions. Although some ROIs repeatedly appeared in the same broad half of the fold-specific rankings, this does not imply the stable rank ordering of individual regions. Accordingly, region-level or network-level attributions based on SG edge weights should not be treated as reliable biomarkers in the present setting. The instability is consistent with previous observations that feature importance rankings in neuroimaging classification can be highly sensitive to the training sample when signal-to-noise ratios are low [26].

This study has a number of limitations.

The results are based on the CC200 atlas only. Different parcellations may yield different relative performance.

All results use cross-validation within ABIDE-I. External validation in ABIDE-II would strengthen conclusions.

We used a single C-PAC pipeline without global signal regression. Different preprocessing choices affect connectivity estimates. The SG computational cost is O (N^2) in ROIs, limiting scalability to finer parcellations.

The pooled ASD group encompasses substantial clinical heterogeneity. Subgroup analyses could reveal patterns obscured in aggregate.

Future work should map CC200 parcels to canonical functional networks, such as the Yeo seven-network parcellation [27], using reproducible voxelwise overlap between the corresponding atlas volumes in a common MNI space.

The ADOS analysis used ridge regression with a fixed regularisation parameter of α = 1.0 and no hyperparameter tuning. Therefore, the negative result applies to this prespecified model implementation, and alternative regression specifications were not investigated.

The tangent space comparator was evaluated using the first 50 CC200 ROIs in atlas order rather than all 200 ROIs. Although this fixed subset was independent of the outcomes and introduced no feature selection leakage, its arbitrary composition may have affected the estimated performance of tangent space connectivity.

This study was not pre-registered. Although this work is a methodological benchmark rather than a confirmatory hypothesis test, pre-registration would have strengthened this study by documenting a priori expectations about the relative performance of SGs in the resting-state setting. All analytical methods, parameter settings, and results for all evaluated methods are reported to ensure transparency.

No a priori power calculation was performed for any analysis. The ADOS prediction analysis (*n* = 193) was particularly underpowered for detecting small effects in high-dimensional data, and the negative result should be interpreted with this limitation in mind.

More broadly, MedLP-HAFB-CLIP demonstrates how learnable, task-adaptive medical prompts can incorporate domain information into a pretrained model for medical image classification [28]. Although that architecture was developed for foetal ultrasound and is not directly transferable to resting-state fMRI, analogous task-adaptive prompting or representation-learning strategies could be explored in future pretrained neuroimaging or multimodal models for brain connectivity classification.

## 5. Conclusions

SGs capture statistically significant but modest diagnostic information in ABIDE-I (57.0%, *p* = 0.001). However, conventional correlation-based representations substantially outperform SGs (∼68% balanced accuracy) and generalise well across sites, even after confound residualisation. These results suggest that SGs’ advantages may be more pronounced in settings with strong task-evoked temporal features and that resting-state ASD classification is better served by correlation-based approaches.

## Figures and Tables

**Table 1:** Participant demographics. *p*-values: independent *t*-test (continuous) and $\chi^{2}$ (sex).

	ASD (*n* = 403)	TC (*n* = 468)	Test
Age (years)	17.1 ± 8.0	16.8 ± 7.2	*p* = 0.661
Sex (M/F)	349/54	378/90	*p* = 0.021
Full-scale IQ	105.8 ± 17.1 (*n* = 376)	111.1 ± 12.1 (*n* = 435)	*p* < 0.001
Mean FD (mm)	0.126 ± 0.143	0.094 ± 0.082	*p* < 0.001
Scan length (TRs)	192 ± 57	195 ± 59	*p* = 0.45

**Table 2:** Primary classification results (10 × 5-fold CV, all methods). All values: mean ± std across 50 evaluations.

Method	Bal. Acc. (%)	AUC	Sens. (%)	Spec. (%)	F1	PR-AUC
Direct (LogReg)	56.5 ± 2.7	0.594 ± 0.029	52.7	60.3	0.529	0.566
Corr Matrix (LogReg)	67.9 ± 3.3	0.743 ± 0.034	65.0	70.9	0.653	0.706
Corr Graph (LogReg)	58.8 ± 3.3	0.618 ± 0.030	53.8	63.8	0.549	0.586
Tangent (LogReg)	60.3 ± 3.5	0.640 ± 0.038	55.8	64.8	0.567	0.590
Elastic Net	57.7 ± 2.6	0.611 ± 0.025	53.4	62.0	0.540	0.588
SVM	54.8 ± 2.7	0.600 ± 0.028	22.1	87.5	0.310	0.571
XGBoost	52.5 ± 3.4	0.533 ± 0.039	41.4	63.5	0.450	0.512
SG (LogReg)	57.0 ± 3.5	0.598 ± 0.036	51.8	62.2	0.530	0.565
Combined (LogReg)	68.0 ± 3.6	0.744 ± 0.036	65.0	71.1	0.654	0.708
Baselines						
Chance (balanced acc.)	50.0	0.500	–	–	–	–

**Table 3:** Site-aware validation: Balanced accuracy (%) for all methods. LOSO: Unweighted mean across sites with ≥20 subjects.

Method	LOSO	Site-Stratified CV
Direct	54.8 ± 7.4	55.0 ± 5.3
SG	55.0 ± 8.1	56.3 ± 4.3
Tangent	58.2 ± 9.9	58.7 ± 3.6
Corr Matrix	66.0 ± 8.9	67.5 ± 3.6
Combined	66.2 ± 7.8	67.3 ± 3.1

**Table 4:** Original and covariate-residualised balanced accuracy for the imaging models. The exploratory phenotype-only model was evaluated separately in 811 complete cases and is not directly comparable with the imaging models evaluated in 871 participants.

Method	Original	Residualised	Δ
Direct	56.5	55.2	−1.3
SG	57.0	55.6	−1.4
Tangent	60.3	58.6	−1.7
Corr Matrix	67.9	66.9	−1.0
Combined	68.0	67.1	−0.9
Phenotype Only (n=811)	59.2	–	–

## Data Availability

ABIDE-I raw imaging and phenotypic data are publicly available from the International Neuroimaging Data-sharing Initiative (INDI) 1000 Functional Connectomes Project at http://fcon_1000.projects.nitrc.org/indi/abide/abide_I.html (accessed on 11 July 2026). C-PAC-preprocessed derivatives (filt_noglobal strategy, CC200 parcellation) are available from the Preprocessed Connectomes Project at http://preprocessed-connectomes-project.org/abide/cpac.html (accessed on 11 July 2026), also mirrored on NITRC and as a public Amazon Web Services S3 bucket, as described by Craddock et al. [10]. All ABIDE-I data are fully anonymised and contain no protected health information, in accordance with HIPAA guidelines and 1000 Functional Connectomes Project/INDI data-sharing protocols; consequently, no separate data use agreement is required, although users must register with NITRC and the 1000 Functional Connectomes Project to obtain access. The original data collection, including clinical/diagnostic assessment, was approved and conducted by the institutional review boards and clinical teams at each of the 17 contributing acquisition sites, as described by the ABIDE consortium [3]. Raw ABIDE-I data are distributed under a Creative Commons Attribution-NonCommercial-ShareAlike licence and the C-PAC-preprocessed derivatives under a permissive BSD-style licence; our use of both datasets for non-commercial academic research complies with these terms. Analysis code is available from the authors upon reasonable request.
